# Splenic Macrophage Activation and Disordered Heme–Iron Metabolism in a Mouse Model of Acute Hepatic Encephalopathy

**DOI:** 10.3390/ijms27052463

**Published:** 2026-03-07

**Authors:** Kanako Tadokoro, Nozomi Ito, Riku Terashima, Kairi Horigome, Kiyoharu Kawakami, Kazuhiko Nakadate

**Affiliations:** Department of Functional Morphology, Meiji Pharmaceutical University, 2-522-1 Noshio, Kiyose 204-8588, Tokyo, Japan; s212052@alm.my-pharm.ac.jp (K.T.); m256206@std.my-pharm.ac.jp (N.I.); s212055@alm.my-pharm.ac.jp (R.T.); s212065@alm.my-pharm.ac.jp (K.H.); k-kawakami@my-pharm.ac.jp (K.K.)

**Keywords:** hepatic encephalopathy, liver failure, splenic pathology, hemosiderin deposition, hematin pigment, macrophage activation, iron metabolism disorder, erythrocyte destruction, ammonium acetate administration

## Abstract

Hepatic encephalopathy is a severe complication of liver failure, traditionally investigated through brain–liver interactions; however, the involvement of extrahepatic organs remains poorly understood. This study examined splenic histopathological changes in a mouse model of acute hepatic encephalopathy induced by ammonium acetate administration, focusing on iron metabolism and macrophage activation. Although conventional hematoxylin and eosin staining revealed no overt structural abnormalities, unstained spleen sections demonstrated abundant black deposits, predominantly in the red pulp. Prussian blue staining confirmed a significant increase in hemosiderin-positive cells; however, a subset of black deposits was iron-negative. Immunohistochemical analyses revealed that these iron-negative pigments were localized within F4/80-positive macrophages and colocalized with heme oxygenase-1 (HO-1), suggesting enhanced heme degradation. Ultrastructural observations further identified electron-dense granules consistent with hematin accumulation in splenic macrophages. Hematological analyses revealed significant reductions in red blood cell count and hemoglobin levels, indicating accelerated erythrocyte destruction. Collectively, these findings demonstrate that acute hepatic encephalopathy induces splenic macrophage activation, accompanied by disordered iron metabolism and hematin accumulation. This study highlights the spleen as a previously underappreciated extrahepatic organ involved in the pathophysiology of hepatic encephalopathy and suggests that splenic heme–iron handling may represent a novel therapeutic target.

## 1. Introduction

Chronic liver disease and its associated complications significantly affect metabolic and immune functions, resulting in pathological alterations in multiple organs [1]. Hepatic encephalopathy is a neurological disorder induced by liver disease and is characterized by a broad spectrum of clinical manifestations, ranging from mild attention deficits to severe coma [2,3].

Hepatic encephalopathy is a significant complication associated with chronic liver disease and liver failure, and its incidence varies according to disease progression and underlying conditions. Approximately 30–80% of patients with chronic liver disease develop minimal hepatic encephalopathy, which, despite manifesting few subjective symptoms, is linked to cognitive decline and a substantial increase in daily life risks [4]. In contrast, overt hepatic encephalopathy occurs in approximately 30–50% of patients with liver cirrhosis, with a notably high incidence among those with severe cirrhosis (Child-Pugh class C) [5]. Furthermore, among patients with acute liver failure, the incidence of hepatic encephalopathy is exceedingly high (70–80%), and severe cases of grades 3–4 are associated with poor prognosis. Moreover, 50–70% of patients experience recurrence within 6 months of the initial episode, resulting in a marked decrease in quality of life (QOL) and increased utilization of healthcare resources [6].

Hepatic encephalopathy manifests as a spectrum of symptoms, ranging from mild manifestations, such as decreased attention and personality alterations (grades 0–1), to severe confusion and coma (grades 3–4) [7]. The primary pathophysiological characteristics include elevated blood ammonia levels, systemic inflammatory responses, gut microbiota abnormalities, and oxidative stress [8]. Notably, ammonia accumulation affects neurotransmission, induces swelling of glial cells in the brain, and contributes to cerebral edema [9,10,11].

The diagnosis of hepatic encephalopathy is primarily guided by the West Haven criteria, which rely on clinical evaluation supplemented by additional diagnostic tools, such as blood ammonia measurement and neuropsychological assessments. Recommended therapeutic interventions include enhancing the intestinal environment through the administration of lactulose or rifaximin. In addition, branched-chain amino acid (BCAA) supplementation and liver transplantation are viable treatment options for this condition. Despite these interventions, hepatic encephalopathy continues to significantly affect the 1-year survival rate of patients, highlighting the critical need for preventing recurrence [12]. Notably, the 1-year survival rate of patients with cirrhosis and hepatic encephalopathy is approximately 42%, with early diagnosis and appropriate treatment playing a crucial role in influencing patient outcomes [13]. The onset of hepatic encephalopathy not only diminishes the patient’s quality of life but also imposes substantial psychological and economic burdens on their families and caregivers. Consequently, the management of hepatic encephalopathy transcends the mere treatment of the condition itself and represents a significant issue affecting the overall well-being of patients and their support networks, necessitating prompt clinical intervention and comprehensive care.

The low 1-year survival rate of patients who develop hepatic encephalopathy and receive treatment may be attributed to its significant effects on extrahepatic organs. However, research on hepatic encephalopathy has predominantly focused on the liver and brain, and studies targeting extrahepatic organs are infrequent. Given that hepatic encephalopathy affects the brain via the bloodstream, it is plausible that it also has a substantial impact on the circulatory system [14]. Notably, damage to the spleen, which is involved in the removal of abnormal red blood cells and plays a role in the immune system, may influence prognosis; however, the pathological effects on the spleen remain unclear [15]. The spleen functions as a blood filter and as a central organ of the immune system [16]. In cases of portal hypertension associated with chronic liver disease, splenomegaly (abnormal enlargement of the spleen) becomes prominent, and a series of pathological conditions occur, including blood congestion, increased destruction of red blood cells, and abnormal iron metabolism [17,18]. Specifically, the accumulation of substances such as hemosiderin, an iron-containing compound produced during the destruction of red blood cells, and hematin can induce increased oxidative stress and inflammatory responses in the spleen, potentially extending to systemic pathological conditions [19,20]. In contrast to hemosiderin, hematin is a product of the oxidation and denaturation of the heme protein in hemoglobin and typically manifests in conditions characterized by severe bleeding, hemolysis, or oxidative stress [21]. Hematin is an oxidized variant of heme, wherein the central iron atom transitions from Fe^2+^ to Fe^3+^. It is known to accumulate in tissues as a dark brown to black-brown pigment. It is rapidly metabolized by macrophages [22,23].

Macrophages in the spleen constitute a group of cells that are integral to the pathogenesis of liver diseases. These activated macrophages recover iron through phagocytosis of red blood cells and modulate immune responses by secreting inflammatory cytokines [24,25]. Moreover, it has been posited that the aberrant activation of macrophages associated with chronic liver disease disrupts systemic immune equilibrium and may contribute to the progression of extrahepatic complications, including hepatic encephalopathy [26,27]. Given the pivotal role of the spleen in blood circulation and immune regulation, pathological alterations in the spleen are likely to significantly influence systemic conditions. For instance, splenic macrophages are responsible for processing hemoglobin following the destruction of red blood cells, and the metabolic byproducts generated during this process (heme, iron, and oxidants) may exacerbate systemic oxidative stress [28,29]. Additionally, blood stagnation and destruction of red blood cells associated with portal hypertension may cause chronic inflammation and disrupt immune homeostasis [30,31]. From this perspective, to achieve a more comprehensive understanding of the pathology of hepatic encephalopathy, it is imperative to conduct a detailed analysis of pathological changes within the spleen, particularly the accumulation of red blood cell metabolic products and their formation processes. However, the current knowledge regarding how morphological features suggestive of activation and the abnormal accumulation of their products in the spleen contribute to this progression remains limited.

The objective of this study was to elucidate the pathological alterations occurring in the spleen as a consequence of liver disease, and to clarify the role of the spleen in this context. Additionally, this study may lay the groundwork for novel therapeutic strategies targeting disorders of iron metabolism and abnormal macrophage activation and serve as a foundation for future clinical applications. This study specifically employed an acute ammonium acetate–induced hepatic encephalopathy model, and the findings should not be directly extrapolated to chronic liver disease. This investigation was designed as an exploratory histopathological study to provide foundational morphological evidence of splenic involvement in acute hepatic encephalopathy model.

## 2. Results

### 2.1. Ammonium Acetate Metabolism and Pathological Changes in the Spleen

**Figure 1:**
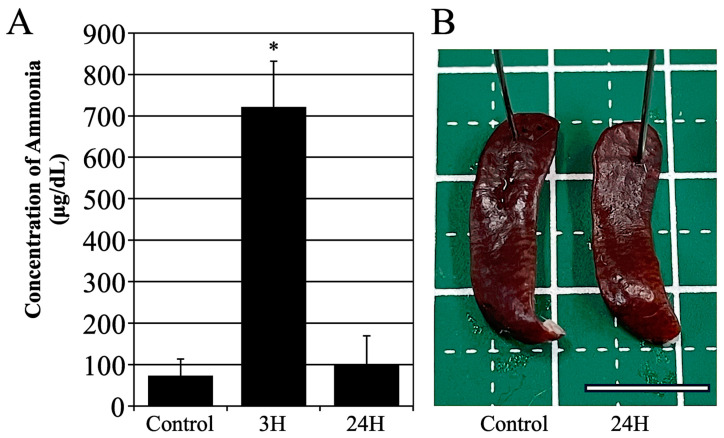
(**A**) Graph of the mean blood ammonia concentration of the animals in the vehicle treatment control group and those treated at 3- and 24-h post-ammonia administration. The data are presented as the mean ± standard deviation. *: *p* < 0.05 compared with the control value. (**B**) depicts the spleen of the control group and the group 24 h following ammonia treatment. Scale bar = 1 cm.

A hepatic encephalopathy model was established by administering ammonium acetate, following a previously documented methodology. The administration of ammonium acetate led to a temporary elevation in blood ammonia levels, observed 3 h post-administration (Figure 1A). A significant increase was observed in the 3 h post-administration group compared with controls (*p* < 0.001). At this stage, the mice were in a comatose state. However, after 24 h, the mice demonstrated a return to normal behavior, and their blood ammonia levels reverted to baseline levels (24 h post-administration, Figure 1A).

Subsequently, a macroscopic anatomical examination of the spleen was performed. As illustrated in Figure 1B, the spleen exhibited a slightly paler appearance 24 h after the administration of ammonium acetate compared to a normal spleen. Nevertheless, as shown in Table 1, there were no significant differences in the size or wet weight.

### 2.2. Hematoxylin and Eosin (HE) Staining

**Figure 2:**
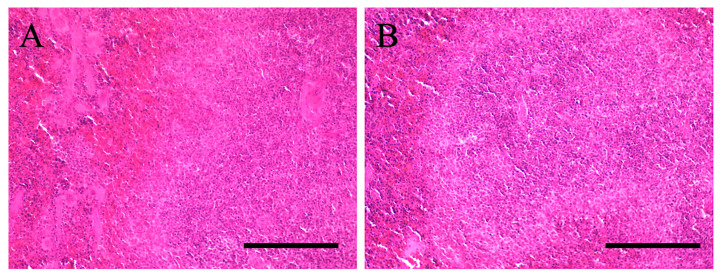
Representative optical images of hematoxylin-eosin-stained spleen tissues are presented for both the control group (**A**) and the group sacrificed 24 h post-ammonia treatment (**B**). Scale bars = 100 µm.

Histological analysis was conducted using hematoxylin and eosin (HE) staining, as depicted in Figure 2A,B. The control spleens exhibited healthy characteristics with uniform staining (Figure 2A). No abnormalities were detected in the red or white pulp, indicating normal functionality. Similarly, the spleens of the hepatic encephalopathy model mice showed no pathological abnormalities (Figure 2B). No significant abnormal structures were observed in the vascular system, suggesting a high likelihood of normal functioning.

### 2.3. Non-Staining Analysis

**Figure 3:**
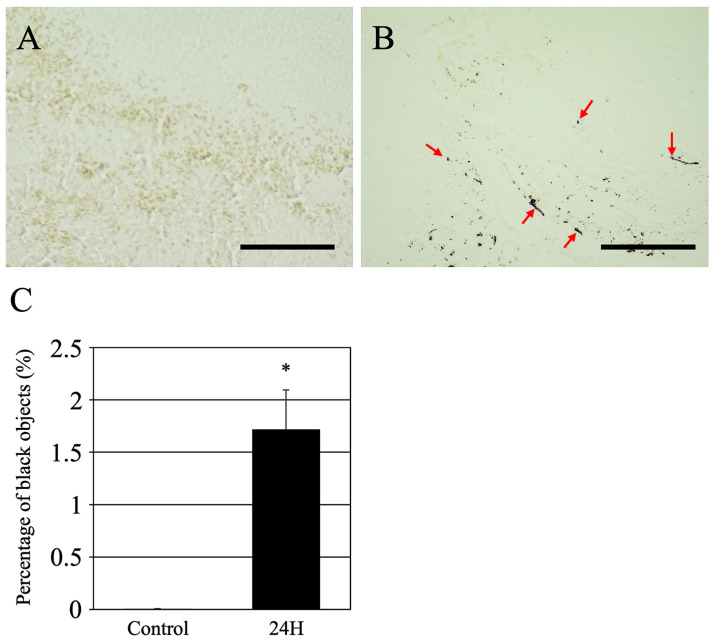
Representative optical images of non-stained spleen tissues are presented for both the control group (**A**) and the group sacrificed 24 h post-ammonia treatment (**B**). Scale bars = 100 µm. Red arrows show the black objects. Graph (**C**) illustrates the proportion of area occupied per unit. The terms “Control” and “24 H” refer to the groups of animals in the vehicle treatment control group and those treated with ammonia for 24 h, respectively. The data are presented as the mean ± standard deviation. A *p*-value of less than 0.05, denoted by *, indicates statistical significance when compared to the control group.

Abnormal pigment deposition is frequently observed in the pathological tissues associated with various disease conditions. In this study, we investigated the occurrence of abnormal pigment deposition in an acute hepatic encephalopathy model. Although pigment deposition can be detected using HE staining, we conducted a comparative analysis with unstained tissue to identify more pronounced alterations (Figure 3A,B). In the control spleens, the tissue appeared almost uniformly colorless (Figure 3A). Conversely, numerous black, punctate, or patchy structures were observed in the group treated with ammonia for 24 h (indicated by red arrows in Figure 3B). These black structures were distributed throughout the tissues, predominantly in the red pulp of the spleen. The quantification of these differences as a proportion of the unit area revealed a significant increase, as illustrated in Figure 3C. A significant increase was observed in the treated group compared with controls (*p* < 0.001).

### 2.4. Fe Staining

**Figure 4:**
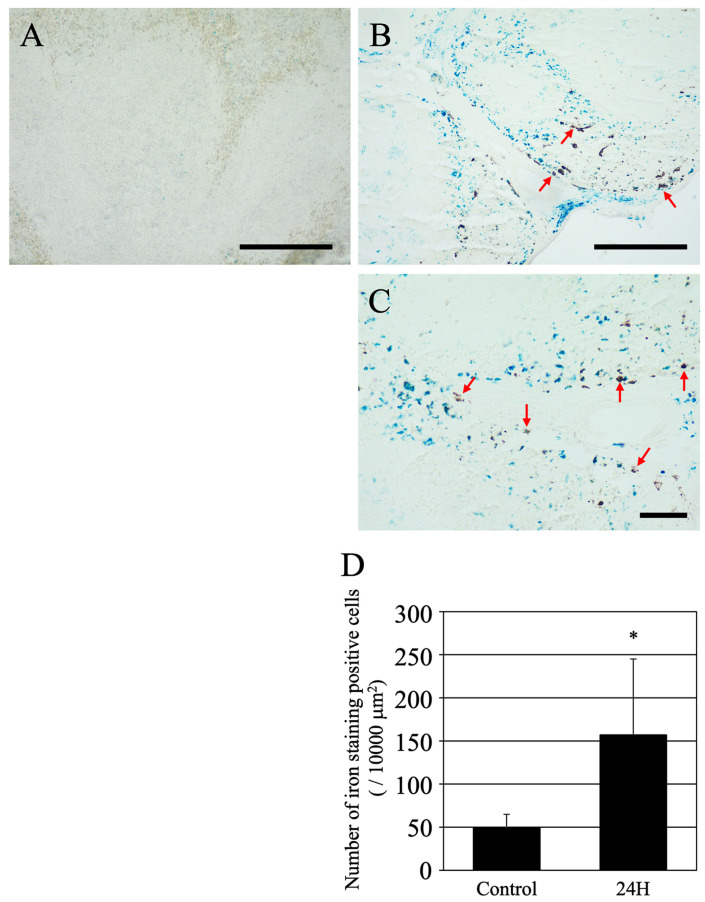
Representative optical images of Fe-stained spleen tissues are presented for both the control group (**A**) and the group sacrificed 24 h post-ammonia treatment (**B**,**C**). Scale bars = 100 µm. Red arrows show the non-Fe-stained black objects. Graph (**D**) shows the number of iron-stained positive cells per unit area. The terms “Control” and “24 H” refer to the groups of animals in the vehicle treatment control group and those treated with ammonia for 24 h, respectively. The data are presented as the mean ± standard deviation. A *p*-value of less than 0.05, denoted by *, indicates statistical significance when compared to the control group.

To ascertain whether the observed black structures were hemosiderin, an iron-containing compound, iron staining was performed (Figure 4A–C). In the control spleen, a minimal amount of iron-positive hemosiderin was detected, as indicated by light blue staining. These cells exhibited faint staining and appeared almost homogeneous and nearly colorless (Figure 4A). Conversely, in the spleens of hepatic encephalopathy model mice, numerous intensely stained blue punctate structures were observed (Figure 4B). Moreover, as illustrated by the magnified views in Figure 4C, there were regions in which iron-positive hemosiderin, denoted by blue staining, did not correspond to the black structures observed (red arrows in Figure 4B,C). Quantification of the number of iron deposits per unit area revealed a significant increase (Figure 4D). A significant increase was observed in the treated group compared with controls (*p* = 0.021).

### 2.5. Immunohistochemical Analysis

**Figure 5:**
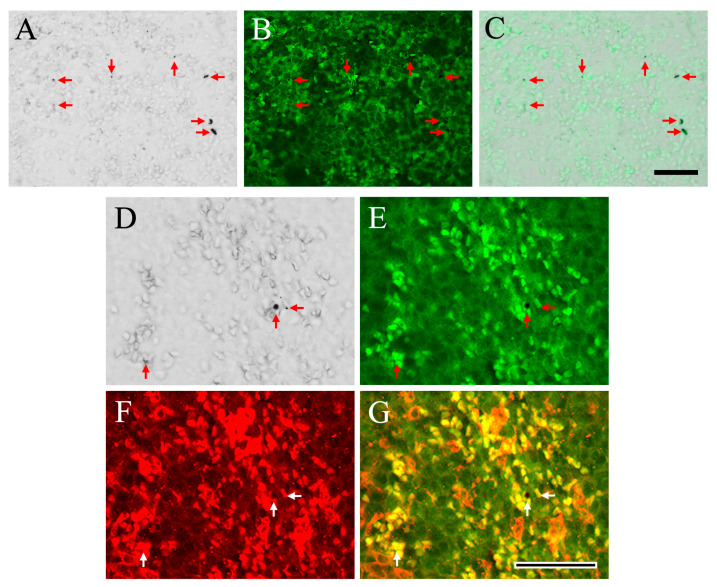
Representative bright-field and immunohistochemical images of spleen tissues are shown. Bright-field images reveal non–iron-stained black structures (red arrows), presumed to represent putative hematin deposits (**A**,**D**). Immunostaining for the macrophage marker F4/80 is shown in (**B**,**E**). Merged images demonstrate that these black structures are localized within F4/80-positive macrophages (**C**). Immunostaining for heme oxygenase-1 (HO-1), a key enzyme involved in heme degradation, is shown in (**F**), and merged images confirm the co-localization of the black structures with both F4/80 and HO-1 (**G**). Scale bars = 50 µm.

After confirming that the black structures were not hemosiderin, we hypothesized that they could be hematin, a degradation product of hemoglobin associated with pathological conditions such as hypoxia [32,33]. Hematin is decomposed and metabolized by macrophages in the spleen. In this study, we employed specific antibodies targeting macrophages to investigate the presence of black structures within these cells (Figure 5). As depicted in Figure 5A, these black structures were visible in the bright field (indicated by red arrows). Upon identification of macrophages using F4/80, a recognized macrophage marker (Figure 5B), it was confirmed that the black structures resided within the macrophages (Figure 5C). Additionally, we explored the association with Heme Oxygenase-1 (HO-1), an inducible enzyme crucial for heme degradation, oxidative stress defense, iron metabolism, and anti-inflammatory functions (Figure 5D–G). Although HO-1 expression is induced in various cell types, it plays a pivotal role in heme degradation within macrophages. The black structures illustrated in Figure 5D were found to coexist with F4/80, the macrophage marker (Figure 5E), and their co-localization with HO-1 was also verified (Figure 5G). These findings suggest that the black structures are localized within macrophages where heme degradation is enhanced. Pigments consistent with putative hematin deposition were observed; however, definitive biochemical confirmation was not performed.

### 2.6. Electron Microscope Observation

**Figure 6:**
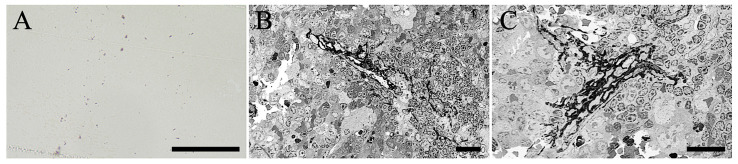
Image (**A**) was captured using an optical microscope of a section with a thickness of 500 nm. Images (**B**,**C**) were obtained using an electron microscope of sections with a thickness of 200 nm. Scale bar in (**A**) = 100 µm and in (**B**,**C**) = 20 µm.

Consequently, we used electron microscopy to determine whether the cells incorporating the black structure were macrophages (Figure 6). The specimens were embedded in epoxy resin and sectioned at thicknesses of 500 nm and 200 nm. Light microscopy of 500 nm-thick sections revealed the presence of black structures, as depicted in Figure 6A. The black structures exhibited a variety of morphologies, including both large and small granules, as well as forms that were nearly rectangular or featured multiple protrusions. These observations are consistent with the morphologies identified using the optical microscope, as depicted in Figure 3, Figure 4 and Figure 5. Electron microscopy of 200 nm-thick sections confirmed the incorporation of black structures within macrophages (Figure 6B,C). Given our primary objective of identifying black structures, we intentionally observed the specimens without electron staining. No significant histopathological alterations were observed in lymphoid cells, which were abundant in the spleen.

### 2.7. Blood Test

In hepatic encephalopathy, iron deposition and hematin production are observed within the spleen, suggesting potential acceleration in the destruction of red blood cells. Consequently, we investigated the alterations in blood characteristics associated with hepatic encephalopathy. Initially, we analyzed the blood cell components (Table 2). These findings revealed a significant reduction in the number of blood cells (RBC) in the hepatic encephalopathy model (*p* = 0.033). Furthermore, hemoglobin (HGB) levels markedly decreased (*p* = 0.041). Although the hematocrit (HCT) showed a decreasing trend, this was not statistically significant. Conversely, while the number of white blood cells (WBC) did not differ significantly, there was a tendency toward an increase. As shown in Table 3, the results of blood biochemical tests indicated elevated levels of liver function markers, specifically aspartate aminotransferase (AST; *p* = 0.011) and alanine aminotransferase (ALT; *p* = 0.029), suggesting impaired liver function.

## 3. Discussion

The present findings were obtained in an acute ammonium acetate–induced hepatic encephalopathy model and should be interpreted within this acute context. Unlike chronic models of liver disease, no splenomegaly was observed in this acute model, suggesting that pigment accumulation precedes organ enlargement. In this study, we conducted an analysis of histopathological changes in the spleens of hepatic encephalopathy models utilizing both light and electron microscopy. Particular emphasis was placed on iron deposition and ultrastructural alterations in macrophages. We observed significant iron accumulation and morphological changes in splenic macrophages. These alterations indicate a systemic inflammatory response and compensatory activation of the reticuloendothelial system associated with hepatic encephalopathy. Notably, the observed increase in iron deposition was likely influenced by accelerated hemolysis and erythrocyte destruction, as well as disruptions in hepatic iron metabolism. The relatively small sample size represents a limitation of this exploratory study.

Hepatic encephalopathy is a neuropsychiatric syndrome arising from diminished hepatic metabolic function and is categorized into acute and chronic forms [34]. The acute form is associated with fulminant hepatitis and progresses rapidly, whereas the chronic form develops gradually with chronic liver disease or portal hypertension advances [26]. Primary pathophysiological factors include impaired ammonia metabolism, systemic inflammation, increased oxidative stress, and alterations in the gut microbiota [35]. The clinical implications of hepatic encephalopathy are expected to extend beyond central nervous system dysfunction, potentially inducing systemic pathologies through alterations in the immune and circulatory systems. However, detailed reports on this are scarce. Previous studies have documented splenomegaly, red pulp expansion, and morphological features suggestive of activation as changes in the spleen during other acute liver failures [36,37]. In addition to these findings, the present study identified microstructural changes, such as the accumulation of iron granules under light microscopy and the accumulation of hematin granules within macrophages at the electron microscopic level. These findings suggest that in acute hepatic encephalopathy, the spleen functions not only as a blood reservoir but also plays a significant role in systemic iron homeostasis and immune response regulation.

Iron metabolism disorders are commonly associated with liver diseases, particularly those characterized by increased iron release because of reduced hepatic hepcidin production, as reported in cases of acute liver failure [38,39]. The observed increase in iron deposition within the spleen in this study may represent an aspect of systemic iron overload and is also hypothesized to result from excessive erythrocyte processing by reticuloendothelial macrophages. Moreover, excessive iron accumulation facilitates the generation of reactive oxygen species (ROS) via the Fenton reaction, thereby increasing local oxidative stress, which may contribute to splenic tissue damage and alterations in immune cell function [40,41]. In the context of systemic immune suppression (immune paresis) observed during acute liver failure, a reduction in lymphocyte density within the white pulp of the spleen has been documented [6,42]. This phenomenon can be attributed to secondary immunodeficiency and the induction of apoptosis following a cytokine storm [43]. The observed splenic alterations may reflect the combined effects of hyperammonemia, systemic inflammation, and possible hypoxia-associated stress. The spleen plays a pivotal role in antigen presentation and immune cell activation, and structural and cellular alterations in the spleen may directly lead to increased susceptibility to infections and poor prognosis. Although changes in lymphocyte counts were not analyzed in the present study, lymphocyte density in the white pulp could not be excluded. This aspect warrants further investigation. The observed splenic alterations may reflect the combined effects of hyperammonemia, systemic inflammation, and possible hypoxia-associated stress.

The pathological alterations in the spleen identified in this study may be direct outcomes of ammonia metabolism in the liver or may be associated with portal hypertension resulting from pathological changes within the liver. Portal hypertension is a complication frequently observed in chronic liver disease. It is characterized by an increase in the hepatic venous pressure gradient, which disrupts blood circulation within the abdominal cavity, thereby increasing the likelihood of blood congestion in the spleen [44,45]. In our previous study, we reported that blood congestion occurs within the liver during hepatic encephalopathy [10]. Consequently, the spleen exhibits pathological splenomegaly, leading to the abnormal accumulation of blood cells and metabolic changes. Specifically, increased red blood cell destruction (hemolysis) occurs, followed by abnormalities in iron metabolism [46,47]. When metabolic disorders of red blood cells become chronic, phagocytosis of red blood cells by macrophages intensifies, during which hemoglobin breakdown products are produced [48]. Notably, hematin is an oxidized pigment derived from hemoglobin formed after red blood cell destruction, and is produced as a result of chronic blood abnormalities [49,50]. Hematin, an insoluble degradation product of porphyrin rings, tends to accumulate locally. The formation of this substance is promoted under conditions of strong oxidative stress, but the detailed mechanisms of its formation and its pathological significance remain largely unclear [51,52]. These findings indicate that the urgent establishment of treatment methods, along with further detailed analyses, is needed to prevent various secondary diseases resulting from increased red blood cell destruction.

HO-1 is an inducible enzyme that degrades heme and plays a crucial role in oxidative stress defense, iron metabolism, and anti-inflammatory processes [53]. HO-1 catalyzes the breakdown of heme derived from hemoglobin and heme proteins, thereby detoxifying harmful heme while generating molecules beneficial to the organism [54]. By converting biliverdin to bilirubin, which possesses potent antioxidant properties, and producing carbon monoxide (CO), HO-1 exerts anti-inflammatory, vasodilatory, and anti-apoptotic effects [53,55]. In this study, a significant increase in hemosiderin was observed in models of hepatic encephalopathy onset, alongside a marked increase in hematin structures. The accumulation of hematin in HO-1 positive cells suggests that, in addition to iron metabolism, the breakdown of heme in red blood cells is enhanced in hepatic encephalopathy models. This indicates that, in cases of liver dysfunction, abnormal red blood cells are rapidly metabolized in the spleen, highlighting the active role of splenic macrophages in providing a “stress-responsive defense” to protect the body. Additional evaluation of ROS-related pathways and iron-regulatory molecules will be important in future investigations. The present study was designed as an exploratory histopathological investigation. Although molecular analyses were not performed, the consistent morphological alterations observed provide a structural basis for future mechanistic studies.

The findings of this study are of considerable significance for a comprehensive understanding of splenic pathology in the context of acute hepatic encephalopathy. Notably, the observed association between iron metabolism disorders and morphological features suggestive of activation not only enhances our understanding of the disease mechanism but also suggests potential therapeutic interventions. The efficacy of iron chelation or antioxidant therapy in mitigating inflammation and oxidative stress in the spleen warrants further investigation in subsequent studies. Additionally, alterations in the spleen are correlated with hematological parameters, such as red blood cell count, hemoglobin levels, and ferritin levels. Clinically, the development of noninvasive methods to evaluate pathology using these indicators is anticipated. These findings should be interpreted as preliminary morphological evidence, and direct functional consequences remain to be elucidated.

However, this study has several limitations. First, the analysis was conducted using an animal model of acute hepatic encephalopathy without direct comparison with the pathological changes observed in human cases. Second, it remains challenging to ascertain whether the observed changes in the spleen are attributable to hepatic encephalopathy itself or systemic alterations associated with acute liver failure. Third, further investigations, such as immunostaining and gene expression analyses, are necessary to elucidate the molecular mechanisms underlying iron deposition and morphological features suggestive of activation. This study did not directly assess functional consequences of splenic macrophage activation. Therefore, the findings should be interpreted as preliminary morphological evidence. Future research should incorporate pathological analyses of human autopsy cases and clinical specimens, evaluation of their relationship with blood iron parameters, and intervention experiments targeting iron metabolism and morphological features suggestive of activation to enable a more comprehensive understanding of the role of the spleen. These studies have the potential to not only enhance our understanding of the pathophysiology of acute hepatic encephalopathy but also contribute to the development of novel therapeutic strategies. This study was designed as an exploratory histopathological investigation. Future studies incorporating comprehensive molecular and oxidative stress markers will be necessary to further elucidate the underlying mechanisms.

The present study was designed as a histopathological investigation of splenic alterations during acute hepatic encephalopathy. While mechanistic pathways such as oxidative stress, iron-handling dysregulation, and inflammatory signaling are plausible contributors, these were not directly assessed and require dedicated molecular studies. Therefore, the current findings should be interpreted as morphological and correlative observations that provide a foundation for future mechanistic investigations.

## 4. Materials and Methods

### 4.1. Animals

Adult male C57BL/6J mice (10 weeks old, 22–25 g; *n* = 4 per group) were obtained from Charles River (Yokohama, Japan) and housed under specific pathogen-free (SPF) conditions (temperature: 22 ± 2 °C, humidity: 55 ± 10%, 12 h light/dark cycle) with ad libitum access to standard food and water. All experimental procedures were approved by the Institutional Animal Care and Use Committee of Meiji Pharmaceutical University (No. 2704, 1 April 2023–2025), and were conducted in accordance with the ARRIVE and NIH Guide for the Care and Use of Laboratory Animals. The sample size was determined based on our previous exploratory histopathological studies using similar models and in accordance with animal welfare reduction principles [10,11].

### 4.2. Experimental Design

According to our previous study [10,11], acute hepatic encephalopathy was induced via the intraperitoneal administration of ammonium acetate (4.5 mmol/kg body weight, Sigma-Aldrich, St Louis, MO, USA) using two intraperitoneal injections with a 15 min interval between injections. The control mice received an equivalent volume of saline. The animals were monitored for neurological symptoms and euthanized after the injection.

### 4.3. Tissue Collection

The experiment was conducted either 3 h or 24 h after the administration of ammonium acetate or saline. Following the induction of deep anesthesia using isoflurane, the mice were euthanized, and the spleens were promptly excised. The spleens were briefly rinsed in ice-cold phosphate-buffered saline (PBS, pH 7.4) to remove surface blood and weighed. Spleens were immersed in 4% paraformaldehyde (PFA) in PB for subsequent histological and electron microscopy analyses.

### 4.4. Light Microscopic Observation and Immunohistochemical Analysis

Tissue samples for paraffin embedding were fixed in 4% PFA for 3 days, dehydrated through a graded ethanol series, cleared in Lemosol (Wako Pure Chemical Industries, Ltd., Tokyo, Japan), and embedded in paraffin. Sections (5 µm) were cut using a sliding microtome (REM-710; Yamato Kohki Industrial, Tokyo, Japan) and mounted on slides.

Hematoxylin and eosin (HE) staining was performed for general histopathology. Hemosiderin was visualized using Prussian blue staining with Berlin Blue solution (MUTO PURE CHEMICALS CO., LTD., Tokyo, Japan), and hematin pigments were visualized as non-stained sections.

For the purpose of immunohistochemical analysis, sections with a thickness of 5 µm were mounted on slides and subsequently hydrated. Antigen retrieval (HistoVT One, Nacalai Tesque, Inc., Kyoto, Japan) was then conducted. Following this, the sections were blocked with Blocking One Histo solution (Nacalai Tesque, Inc., Kyoto, Japan) and incubated with the primary antibodies (1:5000, rabbit anti-F4/80 antibody, FUJIFILM Wako Pure chemical Co, Tokyo, Japan, and 1:2000, mouse anti-HO-1 antibody, Proteintech Japan, Tokyo, Japan) for a duration of 12 h. Subsequently, they were incubated with the secondary antibodies (Alexa Fluor 488 conjugated goat anti-rabbit IgG, and Alexa Fluor 555 conjugated goat anti-mouse IgG, Abcam, Cambridge, UK) for an additional 2 h.

Sections were examined under an optical microscope (BZ-X710; Keyence, Osaka, Japan).

### 4.5. Electron Microscopy

Spleen tissues were cut and fixed in 2.5% glutaraldehyde in 0.1 M phosphate buffer (pH 7.4) for 48 h at 4 °C; rinsed in buffer; and post-fixed in 1% osmium tetroxide for 1 h. Samples were dehydrated through graded ethanol, transitioned to propylene oxide, and embedded in epoxy resin (Epon 812). Sections (500 nm and 200 nm) were cut using a diamond knife on an ultramicrotome (Leica UC6, Leica Microsystems, Wetzlar, Germany) and mounted on glass slides. 500 nm thick sections were examined using an optical microscope (BZ-X710, Keyence, Osaka, Japan); the 200 nm thick sections were examined using an electron microscope (IT-800SHL, JEOL, Tokyo, Japan), and digital images were captured.

### 4.6. Image Analysis

Histological images, comprising 10 images per animal for both the control and HE model groups, were analyzed utilizing ImageJ software (Version 1.54, https://imagej.net/ij/, NIH, Bethesda, MD, USA). The percentage area of pigment deposition was quantified within randomly selected microscopic fields. The extent of pigment deposition in the red pulp was evaluated semiquantitatively by two blinded observers. The threshold in the ImageJ software was established at three times the background value, with any structures exceeding this threshold considered positive.

### 4.7. Blood Biochemical Analysis

Peripheral blood was collected from the tail vein of each mouse (4 mice per group) under light restraint using an animal restrainer (CL-4903, CLEA-Japan Co., Tokyo, Japan). To promote blood flow, the tail was warmed using a heat pad for 2 min before puncture. After disinfection with 70% ethanol, the distal tail vein was punctured using a sterile 27G needle, and approximately 100 μL of blood was collected into heparinized microcapillary tubes.

For manual hemocyte counting, the 50 μL of blood was immediately transferred to EDTA-coated microtubes and gently mixed to prevent coagulation. A hemocytometer (Sigma-Aldrich, St Louis, MO, USA) was loaded with 10 μL of the diluted sample and covered with a glass coverslip. After allowing the cells to settle for 2 min, they were counted. Hematological parameters, including hemoglobin concentration (HGB), white blood cell count (WBC), and hematocrit (HCT), were measured using an automated hematology analyzer (Sysmex XN-1000, Sysmex Corporation, Kobe, Japan) according to the manufacturer’s instructions.

For biochemical analysis, each 50 μL sample was diluted with an equal volume of heparin-supplemented physiological saline. Subsequently, the samples were centrifuged at 1500× *g* for 10 min at room temperature, and the plasma obtained was promptly cryopreserved at −80 °C for subsequent analysis. Plasma ammonia concentration was measured using a Cica-liquid NH3 kit (Kanto Chemical Co., Inc., Tokyo, Japan). Additionally, the concentrations of TP, ALB, AST, ALT, and ALP were determined using appropriate test kits (Wako Pure Chemical Industries, Ltd., Tokyo, Japan).

### 4.8. Statistical Analysis

Data are presented as means ± standard deviations (SD). Statistical comparisons between the control and ammonium acetate-treated groups were performed using Student’s *t*-test or one-way ANOVA with post hoc Tukey’s test, as appropriate. Statistical significance was set at *p* < 0.05. significance. Analyses were performed using StatView statistical software (version 5.0, SAS Institute Inc., Cary, NC, USA).

## 5. Conclusions

In conclusion, this study demonstrates that acute hepatic encephalopathy induces distinct splenic histopathological alterations characterized by enhanced erythrocyte destruction, abnormal iron handling, and macrophage activation. To our knowledge, this is one of the first studies to demonstrate iron-negative pigment accumulation in splenic macrophages in an acute hepatic encephalopathy model. While conventional histology revealed minimal structural disruption, detailed analyses uncovered marked accumulation of hemosiderin and iron-negative black pigments within the red pulp. Importantly, these iron-negative deposits were localized to F4/80- and HO-1-positive macrophages and were ultrastructurally consistent with hematin, suggesting intensified heme degradation under oxidative stress conditions. The concomitant reduction in circulating red blood cells and hemoglobin further supports accelerated erythrophagocytosis in the spleen during acute hepatic encephalopathy. These findings propose a “hematin accumulation hypothesis,” in which excessive heme turnover in splenic macrophages may contribute to iron dysregulation and systemic oxidative stress during acute liver dysfunction. Collectively, our results expand the current brain–liver paradigm of hepatic encephalopathy by identifying the spleen as a critical extrahepatic organ involved in disease pathology. Targeting splenic morphological features suggestive of activation and heme–iron metabolism may offer new avenues for therapeutic intervention in acute hepatic encephalopathy. These findings provide a morphological foundation for future mechanistic and functional investigations.

## Figures and Tables

**Table 1:** Spleen morphological data.

	Length of Major Axis (mm)	Length of Minor Axis (mm)	Thickness (mm)	Wet Weight (mg)
Control	23.43 ± 1.13	5.46 ± 0.13	0.48 ± 0.03	113.14 ± 7.24
24 H	23.14 ± 1.21	5.29 ± 0.11	0.49 ± 0.02	109.57 ± 7.14

The terms “Control” and “24 H” refer to the groups of animals in the vehicle treatment control group and those treated with ammonia for 24 h, respectively. Data are presented as mean ± standard deviation.

**Table 2:** Blood cell test data.

	RBC (×10^4^/uL)	WBC (×10^2^/uL)	HGB (g/dL)	HCT (%)
Control	928.6 ± 38.2	26.1 ± 5.8	14.6 ± 0.9	49.2 ± 2.7
24 H	814.9 ± 42.2 *	29.5 ± 5.4	12.6 ± 1.1 *	47.6 ± 2.2

The terms “Control” and “24 H” refer to the mean blood cell number obtained from the vehicle treatment group and the samples collected from animals 24 h post-ammonia treatment, respectively. The data are presented as mean ± standard deviation. Abbreviations: RBC, red blood cell; WBC, white blood cell; HGB, hemoglobin; HCT, hematocrit. Statistical significance was set at less than 0.05, denoted by *.

**Table 3:** Blood chemistry data.

	TP (g/dL)	ALB (g/dL)	AST (IU/L)	ALT (IU/L)	ALP (IU/L)
Control	4.38 ± 0.31	2.54 ± 0.25	68.33 ±3.56	41.25 ± 4.34	212.33 ± 20.18
24 H	4.19 ± 0.45	2.81 ± 0.52	171.45 ± 6.94 *	62.46 ± 3.56 *	225.23 ± 19.46

The terms “Control” and “24 H” refer to the mean blood chemistry values obtained from the vehicle treatment group and the samples collected from animals 24 h post-ammonia treatment, respectively. The data are presented as means ± standard deviations. Abbreviations: TP, total protein; ALB, albumin; AST, aspartate aminotransferase; ALT, alanine aminotransferase; ALP, alkaline phosphatase. Statistical significance was set at less than 0.05, denoted by *.

## Data Availability

The original contributions presented in this study are included in the article. Further inquiries can be directed to the corresponding author.
